# A Case of Vogt-Koyanagi-Harada Disease Associated With Polycystic Ovary Syndrome

**DOI:** 10.4021/jocmr516w

**Published:** 2011-04-04

**Authors:** Mehmet Kaan Kaya, Burak Turgut, Tamer Demir, Ulku Celiker, Bilgin Gurates

**Affiliations:** a Firat University School of Medicine, Department of Ophthalmology, Elazig, Turkey; b Firat University School of Medicine, Department of Obstetrics and Gynecology, Elazig, Turkey

## Abstract

We report a female patient diagnosed as Vogt-Koyanagi-Harada (VKH) and polycystic ovary syndrome (PCOS). She has diagnosed as VKH with diminished vision, bilateral serous retinal detachment, the signs of fundus fluorescein angiography and the findings of optical coherence tomography. The patient was referred to the gynecology clinic for her complaints as weight gain, hirsutismus and amenorrhea. She has also been diagnosed with PCOS. With oral steroid treatment, visual acuity has improved and the detachments have resolved within a month. VKH disease may be associated with polycystic ovary syndrome. The two conditions may have a common autoimmune pathogenesis.

**Keywords:**

Autoimmune pathogenesis; Polycystic ovary syndrome; Vogt-Koyanagi-Harada

## Introduction

Vogt-Koyanagi-Harada (VKH) disease initially manifests in women in their third and fourth decades of life with prodromal meningeal irritation symptoms, including headache, vertigo, nausea, vomiting, and low-grade fever. Posterior uveitis with exudative retinal detachments and optic disc hyperemia follow the above mentioned symptoms. It is associated with extra ocular manifestations such as dysacousia, tinnitus and alopecia, vitiligo and poliosis due to the involvement of the auditory system and the integument system. VKH disease may recur, typically as an anterior uveitis, and may result in marked visual loss due to the posterior segment complications [1, 2].

Polycystic ovary syndrome (PCOS) is commonly diagnosed in young women with anovulatory infertility, oligomenorrhea or hyper androgenic problems such as hirsutism and acnes. The key underlying abnormality is insulin resistance-hyperinsulinemia in the presence of normoglycemia. PCOS is related to hormonal dysregulation, autoimmune mechanisms and metabolic disturbances such as type II diabetes and atherosclerotic conditions [3, 4]. Here, we present the association of VKH disease and PCOS in a young female. To our knowledge, this association has not been reported previously in the literature.

## Case Report

**Figure 1.**
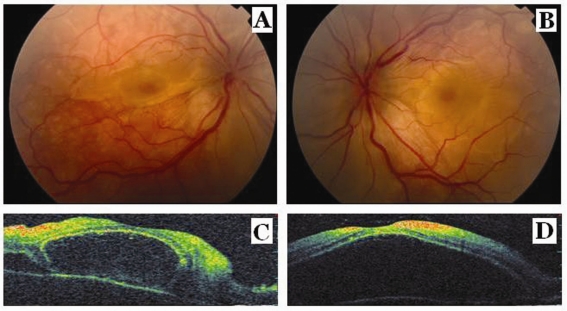
Fundus photographs show serous macular detachment and retinal folds and optic disc edema in the right eye (A) and in the left eye (B). Optical coherence tomography scans show serous macular detachment with several subretinal septas in the right eye (C) and a giant serous macular detachment in the left eye (D) at the presentation.

A 20-year-old female was applied to our outpatient clinic with the complaint of decreased vision in both eyes for a month. Her past medical and surgical histories were unremarkable. Her family history revealed thyroid disease in her mother. The visual acuity in both eyes was revealed 25/60 with normal intraocular pressure. Slit lamp exam revealed 2+ inflammatory cells and flare in anterior chamber in both eyes. The vitreous cavity was clear, and there was no retinal vascular sheathing. Fundus examination disclosed serous retinal detachment and retinal folds, and also optic disc edema in both eyes (Fig. 1A, B). Fundus fluorescein angiography (FFA) OU revealed irregular patches of fluorescence and numerous hyper fluorescent points of leakage at the level of the retina pigment epithelium. The leaking points gradually enlarged and dye accumulated in the sub retinal space. The late-phases FFA showed to diffuse pooling of dye in sub retinal spaces and late optic disc staining in both eyes. Optical coherence tomography (OCT) scans showed serous macular detachment with several subretinal septas in the right eye and a giant serous macular detachment in the left eye (Fig. 1C, D). In this case, a diagnosis of probable VKH disease according to Diagnostic Criteria for Vogt-Koyanagi- Harada [2] was made based on the ocular examination and the typical FFA findings. She was hospitalized for topical and oral steroid treatments (1 mg/kg/day). The patient was consulted by an internist for the etiology other than VKH of the exudative detachment. There was no evidence of any focal neurological deficit. The patient refused the lumbar puncture. A detailed dermatological and audiometric examination revealed no skin or auditory abnormalities. Laboratory investigations showed a normal hemogram and normal liver and kidney function tests. The result of a radiographic examination of the chest was also normal. However, she was referred to the gynecology clinic for her complaints as weight gain, hirsutismus and amenorrhea. Glucose tolerance testing showed the presence of insulin resistance. Pelvic ultrasonography revealed enlarged polycystic ovaries with bilaterally increased stromal thickness and more follicles over 10 per ovary, ranging in diameter from 4 - 9 mm. Her body mass index (BMI) was 26.5. Serum testosterone was elevated (143.8 ng/dl, normal < 80 ng/dl). The diagnosis of PCOS was made by the gynecology clinic and drospirenon + ethinyl estradiol treatment was started. Two weeks after the initiation of oral steroid therapy, sub retinal fluid accumulations in both eyes dramatically decreased and the retinal folds disappeared.

**Figure 2.**
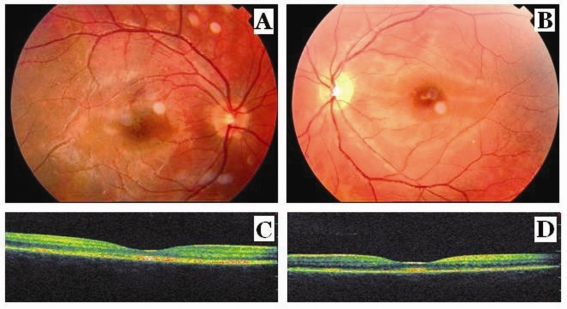
Fundus photographs show complete reattachment of retina, retinal pigment epithelial changes as hyperpigmentation and fibrosis at the macula in the right eye (A) and complete reattachment of retina, retinal pigment epithelial changes as hyperpigmentation and fibrosis at the macula, and the pallor at the optic disk in the left eye (B) after the corticosteroid treatment. Optical coherence tomography scans show the resolution of macular detachment with formation of retinal pigment epithelial irregularities, epi macular membrane and sub foveal fibrosis in the right eye (C) and in the left eye (D) after the corticosteroid treatment.

In the fundus examination at the end of one month of corticosteroid treatment, it was observed complete reattachment of retina, RPE changes as hiperpigmentation and macular fibrosis in both eye and also disc pallor in the left eye (Fig. 2A, B). The best-corrected visual acuity was 20/20 OU; OCT scans showed the resolution of macular detachments with formation of RPE irregularities, epi macular membrane and sub foveal fibrosis in both eyes (Fig. 2C, D). Thus, oral steroid treatment was tapered gradually and ceased at the end of the sixth week of treatment.

## Discussion

Although the exact cause of VKH disease remains unknown, it is thought to be a T-cell-mediated autoimmune processes directed against one or more antigenic components of melanocytes [5]. At present, the diagnosis of VKH disease is usually made based on the clinical findings of prodromal findings of central nerve system followed by a bilateral exudative posterior uveitis. Fluorescein angiography typically reveals multiple hyper fluorescent spots, which coalesce over time and fill the sub retinal space. Ultrasonography shows diffuse thickening of the choroid [2]. Serous retinal detachment and intraretinal fluid accumulation in the outer retina in VKH disease have been demonstrated using OCT [6]. Once the diagnosis of VKH disease is made, an aggressive treatment with high-dose oral corticosteroids, typically in the range of 1 to 2 mg/kg/d should be started. This therapy often results in no visually significant sequel [2]. Vogt-Koyanagi-Harada disease has been reported in association with diseases of autoimmune origin, including thyroid disease, rheumatoid arthritis, and autoimmune polyglandular syndrome type 1 [1, 2]. In addition, it has reported by Yawata et al that the glucose intolerance is present in 55% of patients with VKH disease [7]. In their report, it has been postulated as the possible inflammatory pathogenesis causing both VKH disease and glucose intolerance might have been related. Because the identification of the anti-ovarian autoantibodies and the autoantigens in some patients with PCOS, it has been suggested that an autoimmune mechanism might play a role in the pathogenesis of PCOS in part [8]. In addition, glucose intolerance or insulin resistance which is common finding in PCOS may be a key factor in the pathophysiology of PCOS [9]. Clinical and epidemiological studies have shown that insulin resistance is associated with glucose intolerance [10]. Although VKH and PCOS might be coincidentally found in this patient, a common autoimmune pathogenesis of these two diseases might be taken into account because of glucose intolerance found in both diseases.
